# Joint together: The etiology and pathogenesis of ankylosing spondylitis

**DOI:** 10.3389/fimmu.2022.996103

**Published:** 2022-10-17

**Authors:** Yuehan Xiong, Menghua Cai, Yi Xu, Peng Dong, Hui Chen, Wei He, Jianmin Zhang

**Affiliations:** ^1^ Department of Immunology, Chinese Academy of Medical Sciences (CAMS) Key Laboratory of T Cell and Cancer Immunotherapy, State Key Laboratory of Medical Molecular Biology, Institute of Basic Medical Sciences, Chinese Academy of Medical Sciences (CAMS) and School of Basic Medicine, Peking Union Medical College, Beijing, China; ^2^ Changzhou Xitaihu Institute for Frontier Technology of Cell Therapy, Changzhou, China

**Keywords:** axial spondyloarthritis, hereditary autoinflammatory diseases, Th17 cells, etiology, genetics

## Abstract

Spondyloarthritis (SpA) refers to a group of diseases with inflammation in joints and spines. In this family, ankylosing spondylitis (AS) is a rare but classic form that mainly involves the spine and sacroiliac joint, leading to the loss of flexibility and fusion of the spine. Compared to other diseases in SpA, AS has a very distinct hereditary disposition and pattern of involvement, and several hypotheses about its etiopathogenesis have been proposed. In spite of significant advances made in Th17 dynamics and AS treatment, the underlying mechanism remains concealed. To this end, we covered several topics, including the nature of the immune response, the microenvironment in the articulation that is behind the disease’s progression, and the split between the hypotheses and the evidence on how the intestine affects arthritis. In this review, we describe the current findings of AS and SpA, with the aim of providing an integrated view of the initiation of inflammation and the development of the disease.

## Introduction

Ankylosing spondylitis (AS) is a classic type of inflammatory disease that starts usually with an inflammation in the sacroiliac (SI) joint and ends with the fusion of the spine with a pathognomonic feature called the “bamboo spine”. It belongs to a group of diseases named spondyloarthritis (SpA), featuring inflammation flaring up in the spine, peripheral joints, ligaments, and tendons.

Along with the rapid advancement in medicine, the classification of SpA keeps changing, which in turn inspires as well as limits the way we study it. Initially, AS was recognized as a kind of disease related to rheumatoid arthritis (RA) or rheumatoid spondylitis (1). RA has the hallmark of persistent symmetrical peripheral polyarthritis, including in the hands and feet, and it can affect the cervical spine, causing neck pain and stiffness, while AS usually begins with inflammation in the bilateral lumbosacral joints and less frequently involves peripheral joint inflammation; though, AS was not clearly distinguished from rheumatoid disorders until the discovery of the rheumatoid factor and anti-citrullinated protein antibody (ACPA) (2). In 1974, Moll and Wright grouped non-RA inflammatory diseases into an inter-related family named ‘seronegative spondyarthrides’, including AS, psoriatic arthritis (PsA), reactive arthritis (also known as Reiter’s disease), inflammatory bowel disease (IBD), etc (3). This classification scheme was not widely accepted, yet it did mark off diseases that have considerable comorbidity rates and share signaling cellular pathways in hindsight (4, 5). The classification criteria launched by the Assessment of Spondyloarthritis International Society (ASAS) in 2009 is regarded as a landmark in AS classification, recognizing axSpA as the only entity in comparison to peripheral SpA (pSpA), and the term ‘ankylosing spondylitis’ was fundamentally replaced by ‘radiographical axSpA (r-axSpA)’ (6). These criteria were modified for better classification of the patients and recognition of the medical demand for axSpA that presents unreadable or mild radiographical change (non-radiographical axSpA, or nr-axSpA). However, as a practical standard in classifying patients and avoiding people with unprovoked inflammation in their back but no MRI sign, the term axSpA limits efficient discussion on more closely inter-related constellation of features, including SI damage, HLA-B27, and anterior uveitis that is much more prevalent in former defined AS patients than the other kinds of axSpA or r-axSpA, such as PsA (7). Therefore, AS, as a concept that may or may not manifest on individuals simultaneously, still affords us a useful model to explore the mechanism of pathogenesis.

The pathogenic mechanism of AS remains obscure, but several hypotheses about the initiating process have been proposed. The first one is a direct inference based on ‘self-nonself’ immunology that autoinflammation should be attributed to specific arthritogenic peptides, i.e., aiming to discover a molecular mimicry between the foreign and self-peptide. Despite growing evidence of T-cell clonal expansion in patients, the existence of conclusive common antigens is still challenging to verify (8). Some other theories involve the conformational plasticity of the most critical risk gene, HLA-B27, which has a high prevalence of genetic variants among AS patients (9), as the cause of AS. A series of researches highlights the unfolded protein response (UPR), which is activated during the biosynthesis process of HLA-B27 alleles in the endoplasmic reticulum (ER), due to its consequences of inflammation and autophagy. An error-prone folding process of specific HLA-B27 alleles can instigate endoplasmic reticulum stress (ERS), which activates the UPR and leads to necrotic death and cytokine secretion. Surface-expressed HLA-B27 molecules with unconventional conformation may activate the immune system through the intrinsic HLA monitoring receptor, the killer immunoglobulin-like receptors (KIRs) expressed by CD4^+^ T cells, and natural killer (NK) cells.

Deep understanding of T cell-mediated inflammation has spawned the cytokine blockade strategy, but little is known why this strategy leads to overreaction and underreaction in different situations. In this review, we present organized evidence about how pathophysiological factors contribute to AS development, analyze the consistency among different hypotheses, and make an attempt to reconcile immunological understanding in other fields with findings in AS.

## Cause of AS: Infection or danger?

### The danger theory and AS

A successful immune response would be a quick strong response targeting invading foreign organisms by recognizing its antigenic components *via* immunoglobulin receptors, as the prototypical ‘self-nonself’ theory indicates. Two major modified versions of the theory have been raised to describe the nature of the ligands, known as Charles Janeway’s ‘infectious-nonself’ theory and Polly Matzinger’s ‘danger model’ (10, 11). An adaptive immune response is initiated by the activation of nonclonally rearranged receptors, for instance, the interaction between LPS and Toll-like receptors (TLRs). Janeway proposed these ligands possessing an exogenous nature related to infectious pathogens, termed ‘pathogen-associated molecular patterns (PAMPs)’, while Matzinger held the view that the immune system is more concerned with dangerous conditions, in which chemical substances, named by ‘danger-associated molecular patterns (DAMPs)’, are recognized by the immune system.

With growing knowledge of stimulatory and inhibitory signaling pathways of immunocytes, the canonical model has been expanded. It was known that the activation of naïve T cells requires a primary response when PAMP/DAMPs or cytokine-activated antigen-presenting cells (APCs) simultaneously deliver antigens to TCR (signal one) and costimulatory signals (signal two); while memory T cells only react after re-encountering the same antigen (epitope). However, the bystander activation process is not dependent on antigen encountering. Early in the 90s, Tough et al. have already confirmed that both CD4 and CD8 T cells can be activated with bacterial/viral PAMP but TCR signaling, namely, LPS and poly(I:C) (12, 13). Recent discoveries in animal models such as experimental autoimmune encephalomyelitis (EAE), a model induced by autoantigen to mimic human multiple sclerosis (MS), confirmed that most of the infiltrating T cells are not provided with autoantigen-specificity, while the severity of inflammation could be rescued by knocking down TLRs (14).

**Figure 1:**
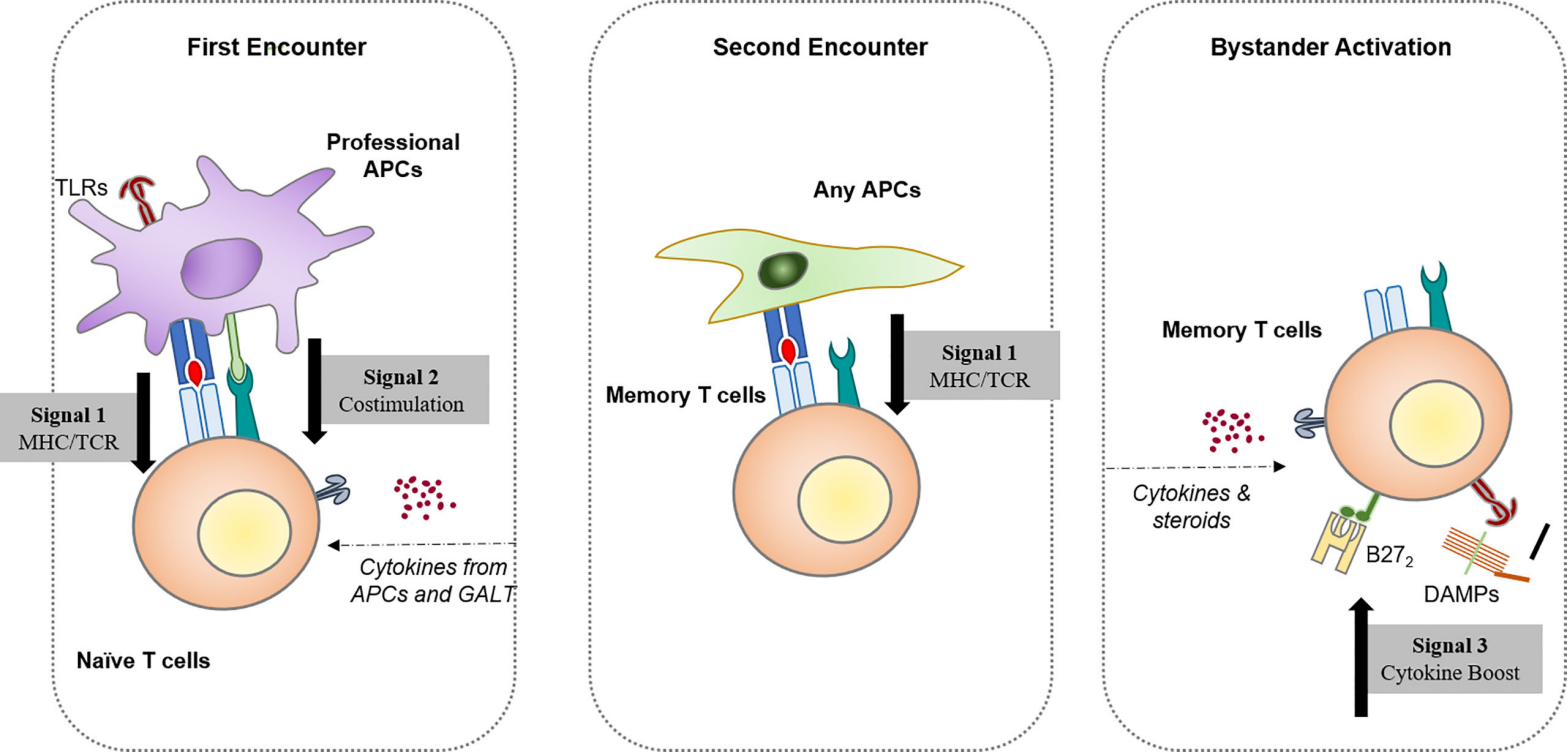
Three different ways resting/naïve T lymphocytes become activated. Activation by the first encounter requires naïve lymphocytes associated with professional antigen-presenting cells, namely, dendritic cells. The second encounter with the antigen is enough to generate a fast, strong response for memory lymphocytes in the resting state. Hypotheses based on the canonical first/second encounter models led to an exploration of potential cross-reactive antigens that elicit spondyloarthritis after pathogen infection. With improved knowledge of the immune regulatory network, the antigen-independent activation of T cells has been identified, which provides a possible pathway for autoreactive T cells that bypasses central tolerogenesis mechanisms.

Perhaps two suppositions could be made on the initial stage of AS: 1) the production of autoantigen has a feature of tissue-specific expression, incurring attack directed by adaptive immunity; or 2) there is a persistent damaged tissue in the axis, releasing signals and priming and bystander-activating immune cells. These signals, either DAMPs or pathogen-associated molecular patterns (PAMPs), can work to offset peripheral tolerance and allow the immune system to attack itself (**Figure 1**).

### Searching for antigens

Molecular mimicry is a common etiological mechanism caused by similar antigenic epitopes shared between self and foreign antigens. Hence, an infection-driven immune response would result in a self-reactive immune system and unresolved inflammation. Early results suggested *Klebsiella pneumoniae*, a Gram-negative bacterium causing pneumonia and sepsis, as a potential candidate. It was first related to AS development due to its fecal carriage and increased serum antibody levels compared to those in HLA-B27-negative patients (15). Then members from Enterobacteriaceae including *Yersinia*, *Salmonella*, and *Shigella* are considered as potential candidates for causing typhoid fever and sequelae of arthritis. The cross-reactivity between HLA-B27 and *K. pneumoniae* was well-studied (16, 17), and underlying molecular mimicry was found, including a homologous hexa- or octapeptide from *K. pneumoniae* nitrogenase (residues 188-193) and HLA-B*2701 (residues 72-77) (18, 19), a tetrapeptide from bacterial pullulanase and collagens (20), and a dodecapeptide from bacterial dipeptidase and collagens (21). The same situation was revealed for *Candida albicans* infection, a dimorphic fungus colonizing the intestinal mucosa, where some research suggests a positive correlation among patients (22) but others did not (23, 24). These conflicting results might be an outcome of the complexity of the intestinal microbiome, where either commensal or pathogenic bacteria generate antibody response, and their function as facultatively causal, consequential, and bystanders to diseases requires arduous work to confirm.

A macroscopic demonstration was given by a recent meta-analysis incorporating 1.3 million cases of AS and 7.6 million healthy controls, which found that there was no significant contribution from bacterial infection (RR [95% CI] = 0.70 [0.10–4.78]), even though previous infections increase the AS risk (25). In comparison, viral infection significantly contributes to AS (RR [95% CI] = 1.43 [1.22–1.66]) (25). HLA-B27 can recognize and present peptides of various viral origins, including influenza A, human immunodeficiency virus (HIV), hepatitis C virus (HCV) (26–29), etc. At the same time, a follow-up study showed that former HPV infection increases the risk of AS (30). Two cohort studies suggested an inverse effect of HIV infection on AS development, suggesting the protective role of an overactivated immune system against viral infection (31, 32). However, even if viral infections increase the susceptibility to AS, it elicits another question about the role of HLA-B27 molecule during this process. Since most peptide-presenting function is inherent to HLA-B27 allotypes (33), these observations suggest that the risk of persistent viral infection is not dependent on a specific antigen but on the pathogenic induction of immune response (unless an epitope expansion occurs).

### Recent advances

Both computational biology and transcriptomics have made great progress in recent years. A host of studies have been conducted with 16S rRNA sequencing and shotgun metagenomic sequencing in search of specific intestinal microorganisms enriched in AS patients. Some bacterial peptides derived from the enriched population are found to match HLA-B27 epitopes according to the prediction tool (the immune epitope database, IEDB) (34, 35). One peptide from *Bacteroides fragilis* mimicking human type II collagen is suggested to be able to interact with HLA-B27 and stimulate the IFN-γ production of PBMC (34). However, it is uncertain whether the pro-inflammatory effect of that peptide depends on carrying the HLA-B27 allotype or heterogeneous inflammatory state among AS patients and healthy controls, the direct binding between HLA-B27 and the peptide appear unlikely in the original study (36). Besides, few consensuses have been reached among other studies: some research confirmed the difference of both microbiota diversity and abundance of specific species, associated with HLA-B27 (37, 38); one suggested that HLA-B27 is not involved in the shift of microbiota during the disease (39), and some discovered an insignificant difference in α-diversity between the AS and HC groups, although specific species correlate with disease activity (40–42). Clonal expansion of CD4^+^ and/or CD8^+^ T cells is also analyzed with immune repertoire sequencing, implying specific antigen-TCR binding in AS (43–45). However, inferring the CDR3 sequences to antigen epitopes is still challenging. Schittenhelm et al. utilized mass spectrometry to enrich HLA-B27 binding peptides with different affinities among HLA-B27 allotypes, but there was only a slight change in abundance (46). Nonetheless, research combining the peptidome and TCR motif brought about some consensual outcomes that disease-related HLA-B27 serotypes bind peptides with C-terminal elongation and specific amino acids are enriched (43, 47, 48). As some studies suggest, this could either be fundamental for the cross-reactive immune response or a consequence of a malfunctioning MHC assembly (48, 49).

In brief, although many research studies suggested a role of HLA-B27 as a direct antigen or mediator of pathogen entry, there are limited clues supporting the causal relation that AS is a sequel to this molecular mimicry process. Instead, HLA-B27 has a potential in providing immunostimulation according to recent investigations for its intercellular and intracellular function. For instance, HLA is under the surveillance of the immunoglobulin-like receptor (KIR) family by the killer cell. KIRs have extraordinary polymorphism and the capability to ‘license’ killer cell function during sensing altered HLA expression and conformers (50). Moreover, it is noteworthy that HLA-B27-related hypotheses could be overrepresented, since only a small percentage of HLA-B27-positive individuals are prone to develop the disease (51); AS patients could be likewise misdiagnosed for carrying HLA-B27, according to a recent study in Spain (52).

## From injury to inflammation

### Enthesitis and synovitis

Our lumbar spine and lower extremities experience high mechanical stress as upright standing species. In the attachment of the tendon or ligament to the bone, the connective part (the enthesis) is easily damaged by stress. This could be one reason why inflammation of entheses, or enthesitis, usually occurs at these sites, particularly in AxSpA (53). Entheses have a similar structure to the growth plate (epiphyseal plate), in which expansion and differentiation of chondrocytes make up a continuous gradient from the uncalcified tendon to the calcified bone. They and their adjacent tissue synovium, named ‘synovio-entheseal complexes (SECs)’ (54), are likely to represent a highly vulnerable part to inflammation. In 1971, John Ball made a very insightful conclusion based on pathological evidence that AS has less destructive erosive synovitis than rheumatoid disease, with unique inflammatory enthesopathy (55). A more modern view of enthesopathy today might be that the repetitive microtrauma as a consequence of body movement could provide a very early signal to initiate SpA and be regarded as a prodromal symptom of it (56, 57). By unloading physical stress in the hind limbs of TNF-overexpressing mice, Jacques and colleagues proved that enthesitis and osteophytes develop only under adequate mechanical stimulation, which was later confirmed in both collagen-induced arthritis (CIA) and collagen antibody-induced arthritis (CAIA) models (58, 59). A discrepancy in body weight bearing leads to different levels of inflammation in different parts of the tarsal and metatarsal bones, reminding us of how AS erodes large joints. However, this model may elucidate only one piece of the genuine disease development in humans. Although the site of enthesitis is found to be connected with bone erosion in humans (60), a cohort covering HLA-B27^+^ subclinical SpA patients revealed that most individuals reported arthralgia, 19% reported inflammatory back pain, and only 5% reported enthesitis (61). The difference between the hypothesis and the findings highlights that enthesitis may be needed for bone lesions rather than inflammation and encourages more planned comparative studies to determine what happens in the early stages of the disease.

### Nonantigenic stimuli from cartilage

Modeled after human RA, the phenotype discovered in both CIA and CAIA mice may still offer some insights into the mechanism of AS development, as efforts to screen cartilage biomarkers for diagnostic and prognostic utility found that peptides derived from type II collagens are correlated with tissue destruction and AS severity (62, 63). It was suspected that type II collagen, the major component of hyaline cartilage, is a potential target antigen during the disease process (64). Similarly, Zou and his colleagues demonstrated that the G1 domain of aggrecan, a large proteoglycan in articular cartilage, is a targetable epitope in AS and RA patients (65, 66). They found that G1 peptide-specific CD8^+^ T cells exist in more than half of the patients, which unfortunately elicited no further exploration. In retrospect, CD8^+^ T cells, even in terms of adaptive immunity, have a questionable role in disease initiation. As shown by the HLA-B27/huβ2m rat model, CD8^+^ T-cell deficiency does not influence the process of SpA initiation (67, 68). Moreover, the pathological imbalance in CD8^+^ T-cell frequency is not influenced by TNFα inhibition therapy, which has proven its efficacy in treating AS patients (69). It was not until Plow and Kollias developed CIA in Rag1^-/-^ immune-deficient mice that the nonantigenic function of type II collagen became obvious (70). Lambert et al. identified a specific fragment derived from type II collagen, named Coll2-1, which can activate synoviocytes to secrete IL-8 (CXCL8) in a TLR-4-dependent manner (71).

In addition to collagen, hyaluronan is an essential component of the extracellular matrix (ECM) and synovial fluid (SF) which is degraded rapidly with aging and inflammation (72). In AS patients, serum hyaluronic acid is also slightly elevated (p = 0.04) and is correlated with some clinical features, such as c-reactive protein (CRP) test, Schober’s test, and the finger-to-floor distance (73). The effect of hyaluronan in inflammation is related to its molecular weight; lower-molecular-weight hyaluronic acid has been reported to facilitate the immune response by binding TLR2 and TLR4 (74, 75), while higher-molecular-weight hyaluronic acid has been demonstrated to be anti-inflammatory through the CD44 signaling pathway (76). Parallel to these findings, it has been shown that TLR1 and TLR2 are strongly expressed in primary human chondrocytes (77). The expression of TNFα and TLR1/2 could even be drastically upregulated by TNFα or TLR1/2 stimuli. Moreover, chondrocytes are able to worsen cartilage degradation by the upregulation of matrix metalloproteinases (MMPs), cathepsin B, and L, which antagonizes the MMP inhibitor (TIMP-1/2) and downregulates ECM proteins (78–80). These findings collectively suggest a very proinflammatory function of chondrocytes during AS development.

### Landscape of innate immunity in AS

Generally, AS is characterized by neutrophil and macrophage/monocyte expansion and synovial infiltration. The hemogram of AS patients shows an elevated neutrophil to lymphocyte ratio (NLR), platelet to lymphocyte ratio (PLR), and monocyte to lymphocyte ratio (MLR) compared to healthy individuals (81–83). All of these features are positively correlated with ESR and CRP, wherein MLR is believed to be a better diagnostic parameter than the others. Immunohistology also demonstrated shared features among non-RA SpA, including AS, PsA, ReA, and JIA, and CD163^+^ M2 macrophage and neutrophil counts were greater in synovial biopsies than in RA and HC (84–86). This specific myelopoiesis pattern could be attributed to different causes, including enhanced monopoiesis/neutropoiesis in the bone marrow (BM) and extramedullary sites, noncanonical precursor differentiation, and the pro-survival effect of cytokines.

In addition to the commonly discussed effect of elevated inflammatory cytokines such as GM-CSF or M-CSF, it has been suggested that monocytes can be generated with an alternative protocol in AS. A traditional paradigm of monocyte differentiation involves sequential binary decisions from granulocyte and monocyte progenitors (GMPs) to monocyte/DC progenitors (MDPs) and finally to common monocyte precursors (CMPs). Since the identification and nomenclature of myeloid cells in the published literature may face a problem of consistency (87), detailed cell identities will not be overly emphasized. It has been proposed by Yáñez et al. that GMP and MDP are derived independently from GMP and are both able to generate so-called Ly6C^hi^ ‘classical’ monocytes in mice, which stands for different means of emergency monopoiesis (88). Two diverse microbial components have been demonstrated to induce different responses. Lipopolysaccharide (LPS), which is conventionally recognized by TLR4 and TLR2 to a degree, stimulates both neutrophil and (neutrophil-like) monocyte differentiation from GMPs, while unmethylated CpG, recognized by TLR9, stimulates monocyte and conventional DC production (88). In line with their observations, increasing evidence suggests a similar process in AS myelopoiesis. In curdlan-injected SKG mice, myelopoiesis is skewed toward GMP, and extramedullary proliferation of GMP is induced by GM-CSF secreted by CD4^+^ T cells and mast cells (89). In humans, monocytes from HLA-B27^+^ AS patients show an adaptation toward a GMP-driven neutrophil-like phenotype when challenged by LPS and cytokines (90).

Moreover, the adaptation of innate immunity, mediated by epigenetic reprogramming, could last a long time. This so-called ‘trained immunity’ has been proven in vaccine- or adjuvant-treated models; for instance, mice treated with β-glucan (a PAMP that activates dectin-1) show increased myeloid cell expansion and enhanced proinflammatory cytokine production from monocytes (91, 92). Mitroulis et al. have demonstrated that hematopoietic stem cells (HSC) from mice treated with β-glucan can preserve a biased differentiation pattern that prefers GMP differentiation after being transferred to irradiated recipient mice for 12 weeks (93). Since AS patients have TLR stimulants including hyaluronan, collagen fragments, and TNFα floating in the SF and peripheral blood (PB), this novel mechanism of innate immunity training can be regarded as a theoretical underpinning of cellular pathogenesis.

It has been well studied that damaged synovium/entheses are able to produce various kinds of inflammatory cytokines and chemokines, engaging in myeloid cell recruitment, activation, polarization, and even osteoclast (trans-) differentiation. Entheseal mesenchymal cells from a mouse model have been demonstrated to secrete CXCL1 and CCL2 (MCP1), which bind CXCR2 and CCR2/4, respectively, in response to mechanical stress (59). *Ex vivo* mesenchymal stem cells (MSCs) from AS patients also show increased CCL2 production (94). Correspondingly, CCR2^+^ M2 macrophages (CD163^+^) are largely increased in PB and synovial biopsies of SpA patients and are correlated with AS disease activity (86, 95–97). This axis participates not only in chemotaxis but also shapes the polarization of macrophages, as CCR2 blockade polarizes macrophages toward an inflammatory M1 phenotype (98, 99). Neutrophil infiltration may also benefit from CCL2/CCR2, as high CCR2 expression and responsiveness are observed in RA patients and an antigen-induced arthritis (AIA) mouse model (100). Research on the neutrophil-specific chemokine CXCL8 shows controversial outcomes, as synovial CXCL8 has been suggested to be elevated only in the SF of RA, aside from other SpA (101, 102), while serum CXCL8 is higher in PsA and AS than in RA (103). These results further suggest a stepwise recruitment protocol of AS wherein neutrophils are recruited by endogenous chemoattractants through vessels and are eventually assembled by TLR stimuli and/or complement components in articulate tissues (104).

### The IL-23/IL-17 axis

The findings of nonantigenic immune activation do not necessarily cancel the importance of adaptive immunity. In contrast, the reduction in the severity of disease in immune-deficient mice confirms a harmful influence of activated lymphocytes, when the IL-23/IL-17 axis has been long suspected to play important role in AS development. In the canonical model of the IL-23/IL-17 axis, the inflammatory IL-17 family as well as other cytokines such as IL-22, TNFα, IL-6 is secreted by Th17 cells, which is induced and maintained by IL-23. A general activation of this axis in AS is proven by an elevated level of IL-23 and IL-17 found in a wide range of AS patients (105–107). Other than Th17 cells, tissue-resident innate immunocytes, such as γδT17 cells and ILC3s, possess a comparable ability in secreting IL-17 and IL-22 (108–110). These cells are the innate counterpart of Th17 cells and have been proven to drive SpA development in IL-23-overexpressing mouse models.

However, after achieving a favorable response rate in PsA and CD, IL-23 blockers failed to show efficacy in treating AS (111–113). Despite evidence from animal models that IL-23-driven IL-17 can stir up enthesitis and SpA (108–110), its causality to AS development has never been settled. According to current evidence, the indeterminate relationship between IL-23 and IL-17 can be explained by redundant IL-17-inducing pathways. Cuthbert et al. firstly reported that the Vδ1 subset of γδT lacking IL-23R is able to secrete IL-17 and IL-22 under anti-CD3/CD28 (mimicking TCR stimulation) or phorbol myristate acetate (PMA) stimulation (114), which provides a substitutional mechanism for IL-23/IL-17 axis. Another potential IL-23-independent activator is prostaglandin E2 (PGE2), a principal mediator of inflammation, which is widely observed to be elevated among AS patients that made the response rate to NSAIDs that inhibits the biosynthesis of PGE2 a criterion for AS diagnosis. Early studies reveal its capability to stimulate IL-17 production and proliferation synergistically with IL-23 *in vitro (* 115, 116), while later an IL-23-independent manner is found on Th17 cells from methylated bovine serum albumin (mBSA)-induced RA model mice as well as RA patients (117, 118). PGE2 is transduced through the prostaglandin E2 receptor 2 subtype (EP2) and the 4 subtype (EP4) expressed on the T cell surface. Under normal conditions, the signaling pathway from PGE2 to the Th17 effector function is regulated in a negative feedback manner, where RAR-related orphan receptor-γ (RORC), the lineage-defining transcriptional factor of Th17, silences EP2 expression by directly binding to its protein coding gene PTGER2 (119). However, the pathogenic Th17 from MS patients shows impaired binding to PTGER2, resulting in an unsuppressed EP2 level and stronger PGE2 signaling. Considering strengthened PGE2 signaling in AS patients compared to HC (120), it remains to be seen that the lacking regulatory capacity of PGE2 signaling could promote AS development by activating Th17 cells. Likewise, a heterogeneity in inflammatory signaling has also been identified in PsA, in that patients with higher skin scores have lower arachidonic acid-derived oxylipins (such as PGE2); on the contrary, those with higher oxylipins are associated with enthesitis (121). This is corroborated by better cutaneous response and limited osteoarticular response to IL-23 inhibitors observed in PsA patients (122). The parallel pro-inflammatory role of IL-23 and PGE2 in both diseases is worthy of further examination.

**Figure 2:**
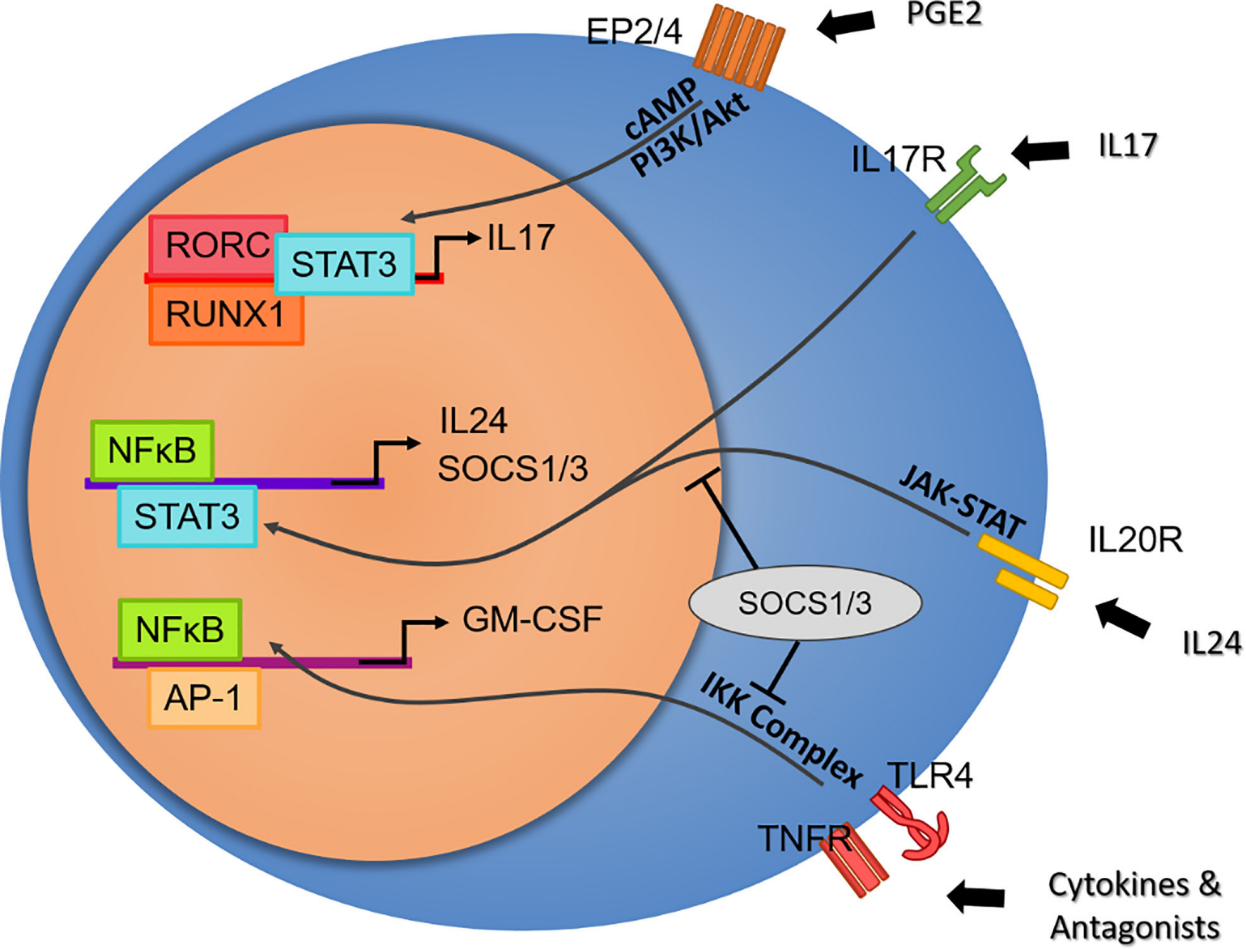
Potential effector axis of Th17 cells independent of IL-23 stimulation. From top to bottom: Prostaglandin E2 plays a critical role in ankylosing spondylitis by activating Th17 cells in an RORC-dependent pathway. Despite its multifaceted proinflammatory effects, IL-17 may overrepresent Th17 function. The paradoxical inflammation induced by the RORC blockade elicits the discovery of the intrinsic negative feedback of IL-17, where the IL-24 autocrine induced by IL-17 regulates cytokine production by SOCS1/3. In contrast, GM-CSF, a secret weapon of Th17, is not under the regulation of RORC. GM-CSF secretion by T cells or nonimmune cells may represent a protocol of the early stage in response to a broad range of tissue damage and DAMPs *via* TLR and TNFR signaling, as well as a particular pathogenic pathway in autoimmune disease.

Searching an inhibitor for IL-17-producing cells brings us to a deeper understanding of IL-17 function. Interesting results are found in screened RORC inhibitors, in that ideally suppressing Th17 function should rescue animal models from developing inflammation. Guendisch et al. first reported that the RORC inhibitor cpd 1 efficiently reduces major Th17 cytokines, including IL-17A, IL-17F, and IL-22, resulting in attenuation in mBSA-immunized antigen-induced arthritis rats (123). Another RORC antagonist, BIX119, has been reported to ablate IL-17 production selectively while sparing IL-22-producing subsets in human SpA- and CD-derived cells (124, 125). Van Tok and colleagues found that using the same compound *in vivo* reduced serum IL-17A, IL-17F, and IL-22 but accelerated the mean onset and worsened the severity in the HLA-B27/huβ2m rat model (126). This paradoxical effect may easily remind us of anti-IL17 treatment in IBD patients (127), but no intestinal inflammation or weight loss was detected in these rats. This counterintuitive problem brings about a deeper examination of the capacity of Th17, in that if it is not a consequence of an imbalanced intestinal immunity, the loss of IL-17 or IL-22 should contribute to the pathogenicity in another way. Chong et al. revealed that the neutralization of IL-17A with a monoantibody abolished the expression of IL-24 and enhanced IL-17F and GM-CSF secretion in autoimmune uveitis and its model (128). They suggested that autocrine IL-17 activates NFκB, leading to IL-24 production, which in turn represses IL-17F and GM-CSF production *via* the SOCS1/3 pathway. This discovery was later supported by a study in EAE mice, where Rorc^-/-^ increases GM-CSF production in CD4^+^ T cells and splenocytes (129). These two elegant studies describe the positive and negative feedback in Th17-cell functioning, where GM-CSF could stimulate macrophages to produce more IL-23 to skew Th17 polarization and increase GM-CSF production, which is suspended when a high concentration of IL-17A activates SOCS1/3 and blocks the expression of GM-CSF (**Figure 2**).

Human Th17 cells are long-lived inflammatory cells, with abundant antiapoptotic gene expression as well as a stem-cell-like phenotype (130). Th17 population is more likely to be self-maintained after differentiation, considering their remarkable capacity to proliferate in resistance to immunosuppression compared with Th0/1/2 (131). In an HLA-B27/Huβ2m mouse model, van Tok et al. showed that anti-IL23R treatment only has a prophylactic protective effect, while anti-IL17A is able to halt inflammation and block new bone formation (132). This may answer the question of why the IL23R polymorphism is associated with AS occurrence rather than its severity (133).

Taken together, disintegrated connective tissues accumulated during the mechanical trauma of the synovium-entheses or cartilage provide enough danger signals to stimulate both innate and adaptive immunity. As a chronic disease, the innate arm can act quickly to injured articulation and sustain a proinflammatory effect by alternating the differentiation program; although there lacks direct evidence of bystander activation in AS, there are alternative ways for the adaptive arm to activate and be activated, while stimulating innate immunity.

## HLA-B27 tuned immunity

### Non-conventional conformers of HLA-B27

In addition to mechanical stress, Polachek et al. discovered an increased risk of enthesitis in PsA with HLA-B27-positive results among 225 patients (134). A similar effect of HLA-B27 occurs in juvenile idiopathic arthritis (JIA) (135). It seems that HLA-B27 alone is capable of perpetuating enthesitis or arthritis in the lower extremities. In the first part of this article, we mentioned that misfolded HLA-B27 is supposed to play a dual role in AS pathogenesis. Conventional MHC molecules are composed of one HLA molecule and a β2m molecule to form a functional MHC heterodimer. The unconventional conformations of HLA-B27 may exist as a free heavy chain (FHC) on the surface or form a homodimer (B27_2_) *via* a disulfide bond on Cys67 (136). The presence of unconventional HLA-B27 has been reported in the intestine and joints of SpA patients (133, 137, 138). They can bind or be recognized by a set of receptors related to the KIR family, including KIR3DL2, leukocyte immunoglobulin-like receptor LILRB2, and the homologous paired immunoglobulin-like receptors (PIRs) of mice (139, 140). Samples from AS patients showed proliferative KIR3DL2^+^ CD4^+^ T cells and NK cells producing significantly more IL-17 than KIR3DL2^-^ cells, which could be reproduced *ex vivo* by coculturing KIR3DL2+ cells with B27_2-_ expressing APCs (141, 142). This reaction could even be rescued by a B27_2_^-^ binding monoclonal antibody, which inhibits the proliferation and survival of KIR3DL2+ cells as well as the production of cytokines *in vitro (* 133, 143). Along with these findings, the idea of HLA-B27 as a KIR stimulant is consistent with the GWAS results, in that the proinflammatory function of KIR-bearing cells is suppressed in people carrying protective HLA-B27 variants or the ERAP1 variant that causes less non-conventional HLA-B27 surface expression (144–146).

### Unfolded protein and endoplasmic reticulum

The formation of an unconventional HLA-B27 conformation is also considered to be inflammatory as a trigger of the unfolded protein response (UPR). MHC class I molecules are assembled in the ER and bind endogenous proteins originating from the cytosol (147). Perturbation during this process may cause protein accumulation and impaired ER function. The conformational change of HLA-B27 is commonly detected *via* conformation-specific antibodies such as ME-1 and W6/32, which can detect folded HLA-B27, and HC10, which is reactive against unfolded or partially folded proteins (148). It has been reported since 2002 that after HLA-B27 heavy chain (HC) synthesis, there can be HC10-reactive peptide accumulation in the ER, while hours later, W6/32-reactive peptides emerge (149). This could be worsened without sufficient β2m or tapasin, a binding protein of ABC transporter TAP. A recent study precisely measured the timing-consumption of different alleles. It takes 3.5 h for susceptible HLA-B*2705 to construct the MHC-peptide complex and 30–90 min for nonsusceptible B*2706 and B*2709 (150). It has also been hypothesized that this prolonged time course may increase the possibility of oxidation at Cys67 and promote B27_2_ formation (149, 151).

UPR orchestration involves one sensor protein, BiP, and three main pathways: inositol requiring 1 (IRE1), protein kinase R-like ER kinase (PERK), and activating transcription factor 6 (ATF6). Finally, the mammalian target of rapamycin (mTOR) and the c-Jun N-terminal kinase (JNK) pathways are initiated and maintain proteostasis through a reduction in protein synthesis, induction of chaperone molecules, and degradation of severely misfolded proteins *via* the ER-associated degradation (ERAD) pathway (152). In HLA-B27/huβ2m rats, enhanced UPR has been detected in intestinal macrophages with increased inflammatory cytokines, including IFNγ and IL-23 (153, 154). A similar difference has been observed in AS patients. Samples from peripheral blood (PB), BM, and SF demonstrated that the expansion of proinflammatory plasmacytoid dendritic cells (pDCs) is accompanied by the upregulation of the PERK pathway (155). Macrophages from PB or SF show the upregulation of UPR as measured by mRNA differential expression, enabling MHC surface expression and the secretion of cytokines, including TNFα and IFNγ (156–158).

In contrast, Ambarus and colleagues found no evidence of UPR in B27-positive macrophages from PB and SF using the same markers as in previous studies (BiP, CHOP, ERdj4) (159). It is contended by Ciccia that autophagy (MAP1LC3A, ATG5/12) but not UPR is activated in AS (160). They investigated intestinal biopsies from AS and Crohn’s disease (CD) patients with both immunohistochemistry and RT-qPCR, and only ERAD ubiquitin ligase SYVN1 was colocalized with HLA-B27 FHCs. In contrast, the findings from the team of Rik Lories support the downregulation of autophagy-related genes (ATG16L1, IRGM, HSP90AA1) in PB (161). Further research revealed autophagy dysfunction with the downregulation of MAP1LC3A and ATG5/12/16L1 in PBMCs, covering all markers used in the previous study (162). This dysfunction is attributed to a decrease in the autophagy mediator lncRNA GAS5.

The function of autophagy is highly contextual, and both increased and decreased autophagy may be involved in disease (163). Autophagy can improve the clearance of pathogenic proteins (164), inhibit apoptotic cell death (165), and obstruct the NLRP3 inflammasome *via* the autophagic removal of NLRP3 activators (166). In the context of autoinflammatory disease, autophagy seems to be more protective. Rapamycin could induce autophagy by blocking the mTOR pathway. Treating HLA-B27/huβ2m rats with rapamycin is able to reduce misfolded proteins by 50% and the severity of disease (167, 168). In humans, it can downregulate the IL-17 and TNFα secretion by PBMCs and inhibit the osteogenic differentiation of fibroblast-like synoviocytes *ex vivo (* 169). With misfolded HLA-B27 accumulating in the ER, autophagy could be induced as a compensatory method for survival but could eventually be disrupted, leading to a remodeled inflammatory phenotype and cell death. This is very likely to occur in antigen-presenting cells, as they are both MHC-bearers and cytokine-producers. In other inflammatory conditions, it has been proven that imbalanced autophagy in dendritic cells and macrophages is extremely pathogenic (170). In contrast, autophagy promotes the production of lysophosphatidylcholine (LPC), an apoptotic inducer and chemotaxin, and the well-known ‘eat-me’ signal phosphatidylserine (PtdSer) (67). These signals promote the scavenging of inflammatory or even antigenic cellular contents. It can be expected that researchers will make better use of autophagy in treating AS. Recent attempts to induce autophagy and apoptotic death in synovial fibroblasts have identified emodin (171) and a combined TNF antagonist and ferroptosis inducer treatment to exert an anti-inflammatory effect (172).

### HLA-B27-related proteins

Beyond HLA-B27, ERAP1, a peptide-trimming enzyme in the ER, is the second strongest risk gene for AS (173). Causing a shift in the peptidome presented by the peptide-MHC complex (174), the pathophysiological effect of ERAP1 is tightly bound to the abnormal assembly of the peptide HLA-B27 (175, 176). In 2013, Kirino et al. discovered that HLA-B51-related BD is associated with ERAP1 alleles in a recessive model based on Turkish and Japanese patients (rs17482078, combined OR [95% CI] = 4.56 [2.88–7.22]), strongly suggesting an independent effect of ERAP1 (177). Following this study, additional SNPs of ERAP1, whether protective or detrimental, were identified among Han Chinese and Turks (178, 179). Kuiper et al. also found that birdshot uveitis, which is prone to affect the HLA-A29^+^ population, was significantly associated with ERAP1, including rs10044354 (OR [95% CI] = 2.07 [1.58–2.71]) and rs2287987 (OR [95% CI] = 2.01 [1.51–2.67]) (180). Given the association with MHC-I risk genes, these diseases were classified by McGonagle et al. into a unified group of ‘MHC-I-opathy’, which provided a good reason for us to explore the commonality in ER dynamics other than the particularity of HLA-B27 (181). It has also been suggested that ERAP1 could intervene in the cleavage or shedding of cell surface receptors of inflammatory cytokines, which after cleavage bind to cytokines without inducing intracellular signals and thus exhibit an inhibitory function. Cui et al. first found that the level of membrane-associated TNF receptor 1 (TNFRI) is negatively correlated with ERAP1 expression without direct protein–protein interactions, suggesting that ERAP1 may assist TNFRI sheddase function (182). That sheddase was later identified as the tumor necrosis factor-alpha-converting enzyme (TACE, or ADAM17) (183). However, several clinical studies verified an increased level of soluble TNFRI in both AS and RA, correlating with ESR and CRP, while it decreased after infliximab or etanercept treatment (169, 184). This makes ERAP1 unlikely to be detrimental by reducing decoy receptors to amplify inflammatory signals.

Collectively, mutations in ERAP1 and HLA-B27 seem to be constantly monitored by a proteostasis network composed of the intracellular UPR, which is meant to correct abnormal folding but could ultimately become apoptotic and proinflammatory, and be subjected to intercellular surveillance by NK cells *via* the surface MHC sensor and KIR family. All of these aspects place accumulative stress on the entheseal or synovial tissue and are probably shared by other MHC-I-related diseases. This presumed mechanism may also evoke an idea of AS treatment by downregulating MHC-I expression in the opposite way as the intrinsic ability of immune escape in tumor cells.

## The potentiality and actuality of gut-joint migration

### Comparison between IBD and AS

Up to 50% of AS patients have subclinical intestinal inflammation, while approximately 10% develop overt IBD, which remarkably resembles CD (185). CD and AS share indisputable similarities. They share many gene variants that are either protective or susceptible, including IL-23R, ERAP1, NOD2, CARD15, etc. Some bacteria, such as *Ruminococcus gnavus*, have been confirmed to be involved in the dysbiosis of both of them (186–188). Enhanced T-cell maturation occurs even in the noninflamed part of the intestinal mucosa from SpA patients, with an increase in the number of lymphoid follicles, CD11c^+^ dendritic cells, CD68^+^ macrophages, and CD11a^+^ pan-lymphoid cells. The pathological features of the AS intestine resemble the early phase of chronic CD, including mixing infiltrating cells and villous atrophy (189–191). However, they differ in several aspects. Recruited CD14^+^ macrophages accumulate in the LP (lamina propria) of CD, where the proinflammatory microenvironment polarizes half or more macrophages toward the M1 phenotype (192–195). At the same time, macrophages in the AS intestine are mainly tissue-resident and undergo M2 polarization (95, 193). What’s more, CD has an increase in regulatory T cells (Tregs) in the intestinal mucosa but a huge decrease in the PB, suggesting a decompensation in the immunotolerogenesis of CD (196, 197), while AS brings no significant change of Tregs count of PB, but an upregulation in LP comparing to HC (198, 199). As a result, IL-17-producing cells controlled by Tregs are more polarized and proliferative in CD than in AS (199–202), leading to the impaired barrier function reflected by Paneth cell malfunction. Overall, CD and AS are not the same diseases even in the local intestine.

The gastrointestinal system is highly complicated, and it could be problematic to expect a stimulus to penetrate the intestinal homeostasis and affect specific aspects of articulation. Taurog et al. reported that rats cultured in germ-free conditions cannot develop arthritis or colitis until the gut microbiome is reimported (203). However, considering that even pulse dosing of antibiotics could impact the expansion and development of intestinal immunocytes (196, 197, 204), it could be deduced that a germ-free environment impedes the maturation and proliferation of intestinal lymphocytes, comprising up to 20% of total lymphocytes in the body (205). Many studies have implied that intestinal microbiota leads to local immune dysregulation through its metabolites and the breakdown of the intestinal barrier, of which the stimulus is transduced thoroughly to articulation through the immune system.

### Gut-joint chemotaxis

The migration hypothesis holds the same rationale as the idea of bacterial infection-driven AS, in that there is spatial proximity between the sacroiliac joint and the draining lymph nodes located in the lower gastrointestinal tract and the pelvic floor. Cellular identification provides much evidence that cells expressing gut-specific markers are present in inflamed joints. For example, Ciccia et al. identified a group of classic monocytes (CD14^++^CD16^+^) in the synovium expressing CCR9, which directs gut homing under homeostatic conditions, suggesting that gut-derived monocytes participate in AS development (206). The gut could be the major source of circulating antigen-experienced T cells that could be activated in the joint, and CCL20 could be the most important chemokine in this process. Ridley and colleagues reported that KIR3DL2^+^ CD4^+^ Th17 cells expressing gut-homing CCR9 are expanded in the PB in patients with AS (142). They predominantly express CCR6 (207), and its only ligand, CCL20, has been shown to be elevated, particularly in the SF, in AS and RA patients (155, 208). CD14^+^ myeloid cells isolated from human enthesis tissues and adjacent bones have been shown to be the primary CCL20 producer after *ex vivo* induction by LPS and IFNγ (209). In addition, tendon stromal cells also have the capacity to secrete CCL20 after IL-23 overexpression (210). The migration of mucosal-associated invariant T (MAIT) cells, which travel around the PB and barrier tissue, is also largely dependent on the CCL20–CCR6 interaction (211–213). As a consequence, Gracey et al. found a reduced frequency of MAIT cells in blood but an increased number of IL-17^+^ MAIT cells in the SF (214), while Toussirot et al. observed that IFN-γ^+^/IL-17A^+^ MAIT cells were increased in the PB of AS patients (215). Interestingly, resident memory CD8^+^ T cells (named CD8^+^ TRM and marked by CD8^+^CD69^+^CD103^+^) were also found to expand in both the inflamed mucosa and PB of HLA-B27^+^ SpA patients, secreting IFNγ (216). They were believed to never leave the tissue in which they reside; however, newly reported evidence suggests that they have the potential to leave their resident tissues, which is called ‘retrograde migration’, and produce circulating effector cells (217). Since few studies concern their migration in response to inflammation, their migration could be CCL20-CCR6 dependent based on discovery in tumor-infiltrating CD8^+^ TRM, and the cytokine pattern in articulation as hereinafter described (218, 219). TCR repertoire sequencing from AS patients has already identified an oligoclonal expansion shared by the intestine and joints, expressing cytokines such as IFNγ, IL-10, and TNFα upon *ex vivo* stimulation (220). A more direct and decisive model is needed, similar to that established by Duc and colleagues in the MS model, whereby disrupting gut homing, they prevented inflammatory cells from getting primed and activated, therefore protecting the mice against developing EAE (221).

### Joint retention of cells from the gut

Chemotaxis is only one aspect of cell recruitment; the other aspects of adhesion and retention require the interaction of adhesion molecules expressed on high endothelial venules (HEVs) or synovial tissues during this step (222). By blocking the binding between integrin and adhesion molecules, anti-integrin biologics prevent immune cells from extravasating and reaching inflammatory sites, to the benefit of both clinical treatment and research. Natalizumab and vedolizumab are two popular anti-integrin biologics used in treating IBD: vedolizumab selectively antagonizes integrin α4β7 while natalizumab blocks α4β1 and α4β7 by binding the α4 subunit (223, 224). With a similar mechanism of action, vedolizumab but not natalizumab is associated with SpA, causing sacroiliac arthritis or arthralgias, particularly as *de novo* cases (225–227). This leads to the conjecture that the additional blockade of the α4β1 of vedolizumab, which plays an important role in ileal homing (228), deflects immunocytes towards articulation. Inflamed joints have abundant ligands for α4β1-binding, including VCAM-1, ICAM-1, and fibronectin. VCAM-1 and ICAM-1 are already proven to be elevated significantly under the inflammatory situation in humans and mice (229–233). The RGD motif containing the three amino acids Arg-Gly-Asp presented in the classical integrin ligand is also found in several ECM proteins, including unraveled fibronectin fragments from the inflamed cartilage (234). In OA patients, fibronectin fragments have been confirmed to interact with chondrocytes, as chondrocytes from the inflamed tissue specifically express α2β1, α4β1, and α6β1 to bind these ECM proteins (235). Fibronectin controlling T cell recruitment is also revealed in the human dermis recently (236).

**Figure 3:**
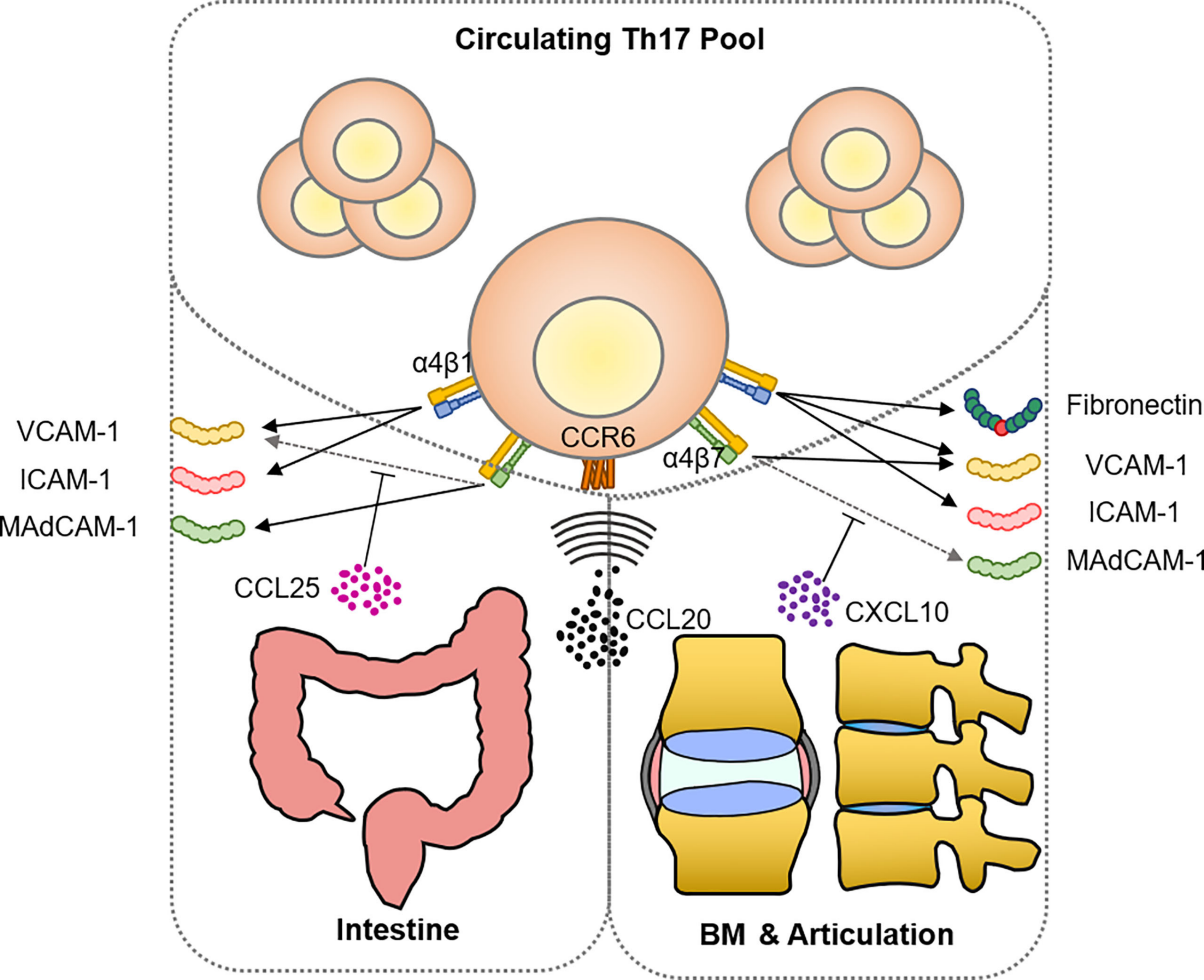
Shared mechanism of recruitment between intestine and articulation in AS. The ligand-receptor pair CCL20-CCR6 constitutes the predominant chemoattraction route of Th17 targeting both intestine and inflamed articulation. Emerging evidence has shown that both tissues express compatible adhesion molecules including VCAM-1, ICAM-1, and MAdCAM-1 for integrin binding, allowing Th17 cells to enter the inflammatory site. Mechanical stress remodeled ECM provides not only T cell stimulant, as the aforementioned hyaluronan and collagen fragment, but also fibronectin fragments that are able to bind and stimulate Th17 cells *via* integrin α4β1.

Besides integrin α4β1, α4β7 is also shared by joint-homing and gut-homing. Mucosal vascular addressing cell adhesion molecule 1 (MAdCAM-1) preferentially interacts with integrin α4β7, directing lymphocyte traffic to intestine. Previous studies shows a putative role for MAdCAM-1 and α4β7 in mediating the BM-homing of hematopoietic cells (237). Ciccia et al. discovered α4β7-expressing ILC3 existing in the intestine, PB, and BM from AS patients (238). A recent finding brings a new perspective to this problem, suggesting that the conformer switch of α4β7 is controlled by CCL25-CXCL10 (239). The CXCL10-activated integrin selectively binds to VCAM-1, while CCL25 induces an extended conformation that binds MAdCAM-1. Correspondingly, the expression level of CCL25 and CXCL10 shifts between the intestinal epithelium and synovium (101, 240). This novel mechanism may further strengthen the ability for T cells to switch their target from gut to joint, utilizing the same integrin binding to different adhesion molecules (**Figure 3**). This deduction is partially confirmed by new findings. For example, Qaiyum et al. discovered that mature CD8^+^ T cells enriched in the SF of AS have a distinct expression pattern of integrins, including α1, αE (CD103), β1, and β4 (of course, they did not bring the universally expressed α4 into the comparison) (241).

Combining clinical evidence and characteristics of immunocytes plus inflamed tissues, a growing body of evidence has provided a framework of the cytological gut-joint axis, which strengthens the idea of gut microbiota-driven disease. Though the synergism and antagonism that exist between intestinal bacteria and the crosstalk with the intestinal immune system remain difficult to investigate, it can be expected that the maneuver of gut-derived cells through recruitment and retention would become a promising target for AS intervention, as it has shown potential in the treatment of MS (221, 242).

## Conclusion

In summary, evidence suggests that there is a radial network in AS pathogenesis, where IL-17-producing T cells play a central role in sensing danger signals and amplifying immune response. On the one hand, the mechanical damage of articulation provides abundant T cell stimulants that may bypass antigen encountering to bystander-activate immune response, yet further investigation on bystander activation in AS is much required. On the other hand, the molecular mechanism of HLA-B27 misfolding and non-conventional presentation becomes apparently associated with immune activation. Although it could still be inaccurate to assert that the AS is not driven directly by any infection, no certain arthritogenic peptide nor molecular mimicry matches have been identified from benchside to bedside. We already know that inflamed joints have a large number of stimuli of TLR and other innate receptors, and that innate immune cells are not only able to respond but also maintain an inflammatory immune memory, which gives weight to the role of non-immune and innate immune cells in autoinflammatory disease. Similarly, alternative pathways have been identified by researchers besides the IL-23/IL-17 axis, where non-immune and innate immune cells are involved through the upstream inducer PGE2 and downstream effector GM-CSF. Furthermore, the transportation between the gut and the joint, through chemotaxis and adhesion, is very clear and shall play an important role in relaying influence from microbiota to autoinflammatory disease.

Nevertheless, we are still trapped in a framework dominated by adaptive immunity, while various pivotal aspects of this protocol remain to be discussed. For example, peripheral tolerogenesis is not emphasized as an equal measure, which could be more important in explaining why many HLA-B27-positive people remain healthy. In addition, bone homeostasis, which regulates the balance of osteoporosis and ectopic ossification, is rarely mentioned, along with other adjacent tissues, since every part of our bodies should be considered as one piece of the immune system. With scientists’ endeavors to decipher this disease over the years, we are finally brought to the stage where the great majority of issues have been identified, although they are not fully understood. This enables us to join every piece of knowledge together to better assist patients and embrace the nature of the complexity of how the body works.

## Author contributions

YHX: Drafting the manuscript. MC: Conception and design of study. YX, HC, and PD: Revising the manuscript critically for important intellectual content. JZ and WH: Supervision, Writing-Reviewing, and Editing. All authors contributed to the article and approved the submitted version.

## Funding

This work was supported by the National Natural Science Foundation of China (82071791, 31970843, 81972866 and U20A20374), the CAMS Initiative for Innovative Medicine (2021-1-I2M-005 and 2021-1-I2M-035), and the CAMS Central Public Welfare Scientific Research Institute Basal Research Expenses (2018PT32004, 2018PT31052 and 3332020035).

## Conflict of interest

The authors declare that the research was conducted in the absence of any commercial or financial relationships that could be construed as a potential conflict of interest.

## Publisher’s note

All claims expressed in this article are solely those of the authors and do not necessarily represent those of their affiliated organizations, or those of the publisher, the editors and the reviewers. Any product that may be evaluated in this article, or claim that may be made by its manufacturer, is not guaranteed or endorsed by the publisher.
